# Supplementary material for: TMT-Based Proteomics Analysis of Senescent Nucleus Pulposus from Patients with Intervertebral Disc Degeneration
Source: Int J Mol Sci. 2023 Aug 26;24(17):13236. doi: 10.3390/ijms241713236 (PMC10488253; doi:10.3390/ijms241713236)
Supplement: Supplementary file 1 [file ijms-24-13236-s001.zip › ijms-2554560-supplementary.pdf]

Supplementary materials

Supplementary Figure S1

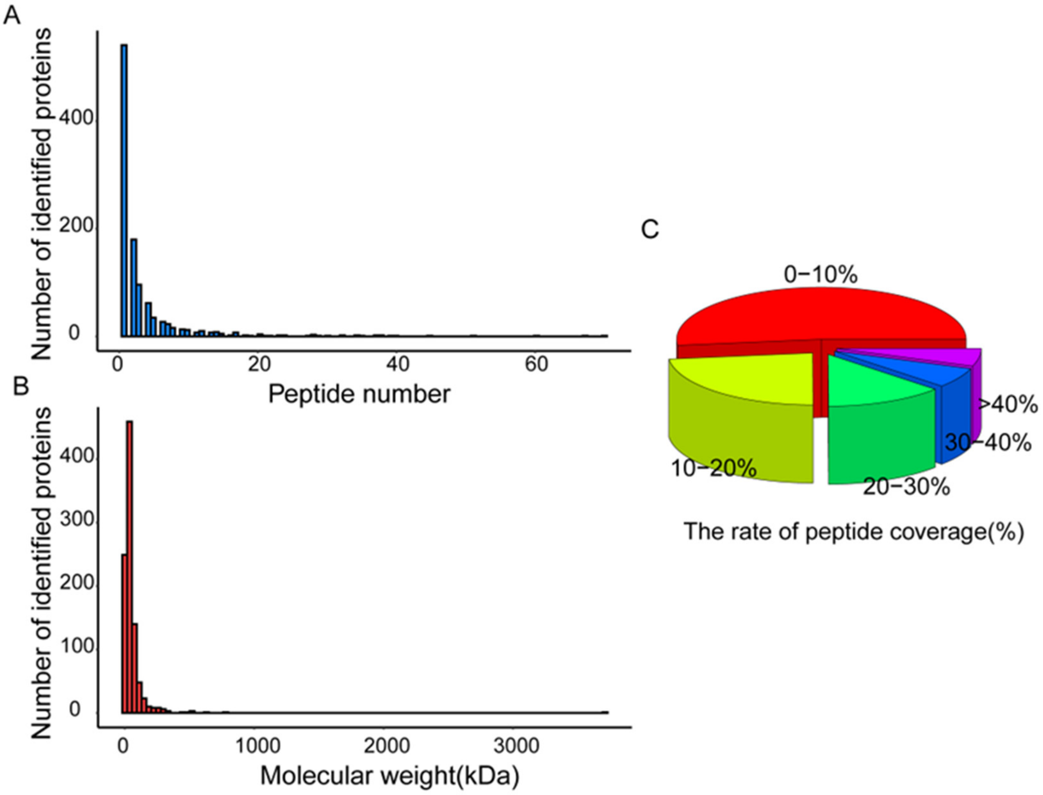

Figure S1. Distribution of peptides, molecular mass distribution, and peptide sequence coverage. (A) Distribution of the number of peptides for each qualitative protein. (B) The number of proteins corresponding to different molecular weights. (C) Peptide coverage rate (%).
